# Headache and wine. Are all wines the same?

**DOI:** 10.1186/1129-2377-14-S1-P32

**Published:** 2013-02-21

**Authors:** S Diaz-Insa, A Rodrigo, YM Pamblanco, L Lacruz, A Soler, C Guillen

**Affiliations:** 1 Headache-Neurology Unit, Spain; 2 Neurology Unit, Spain

## Objectives

Classically, most migraineurs refer some relationship between drinking wine and headache. There are few previous studies confirming this. Our aim is to confirm (or not) this relationship and find out if all kind of wines have the same effect producing headache.

## Methods

Based in previous data we have designed a simple and structured questionnaire. All patients attending a headache clinic (one day/week) during 6 months have been required to fill it. Age, gender, headache type (IHC-2004 criteria) are recorded. Questions referring to usual intake of wine, wine producing headache, kind of wine (red/white/sweet/cava-champagne) were asked. Questions about other alcoholic beverages producing headache and hangover headache also were presented. In this paper we describe the Results.

## Results

397 patients filled the questionnaire. Mean age 44.4 years, 79.6% females. Mean headache days, 11.5 per month. Migraine was the commonest headache type (74.8%). Just 9.1% drink wine usually. 166 patients (41.8%) affirm drinking wine produces headache. Some interesting differences were found between headache types and migraine subtypes: 55.6% of chronic migraine vs just 19% of MAura patients. Patients referring headache due to wine conssumption (most with little quantity) appoints red wine in 60%, cava-champagne 47%, white 36% and sweet 32%. Most of them have stopped drinking wine due to this headache-producing problem. Other alcoholic beverages (mostly high-alcohol-degree) induces headache in 26.7%, 4/5 of them having also wine-induced headache. Hangover headache was recognised by half of the patients along their lifetime, but just 30% of them referred this headache to be similar to their usual headache.

## Conclusions

Headache due to wine conssumption seems to be frequent, at least in migraine patients. Not all kinds of wine are referred as headache inducers, there’s some special sensitivity to different wine types in different patients. Hangover headache feels different to usual headache by the majority.
